# Construction of microbial platform for an energy-requiring bioprocess: practical 2′-deoxyribonucleoside production involving a C−C coupling reaction with high energy substrates

**DOI:** 10.1186/1475-2859-11-82

**Published:** 2012-06-15

**Authors:** Nobuyuki Horinouchi, Takafumi Sakai, Takako Kawano, Seiichiro Matsumoto, Mie Sasaki, Makoto Hibi, Jun Shima, Sakayu Shimizu, Jun Ogawa

**Affiliations:** 1 Division of Applied Life Sciences, Graduate School of Agriculture, Kyoto University, Kitashirakawa-oiwakecho, Sakyo-ku, Kyoto, 606-8502, Japan; 2 Tokyo Laboratory, Yuki Gosei Kogyo Co., Ltd., 3-37-1 Sakashita, Itabashi-ku, Tokyo, 174-0043, Japan; 3 Industrial Microbiology, Graduate School of Agriculture, Kyoto University, Kitashirakawa-oiwakecho, Sakyo-ku, Kyoto, 606-8502, Japan; 4 Research Division of Microbial Sciences, Kyoto University, Kitashirakawa-oiwakecho, Sakyo-ku, Kyoto, 606-8502, Japan

**Keywords:** Energy-requiring bioprocess, Energy-recycle, ATP, Baker’s yeast, 2′-deoxyribonucleoside, Aldolase, Deoxyribosyltransferase

## Abstract

**Background:**

Reproduction and sustainability are important for future society, and bioprocesses are one technology that can be used to realize these concepts. However, there is still limited variation in bioprocesses and there are several challenges, especially in the operation of energy-requiring bioprocesses. As an example of a microbial platform for an energy-requiring bioprocess, we established a process that efficiently and enzymatically synthesizes 2′-deoxyribonucleoside from glucose, acetaldehyde, and a nucleobase. This method consists of the coupling reactions of the reversible nucleoside degradation pathway and energy generation through the yeast glycolytic pathway.

**Results:**

Using *E. coli* that co-express deoxyriboaldolase and phosphopentomutase, a high amount of 2′-deoxyribonucleoside was produced with efficient energy transfer under phosphate-limiting reaction conditions. Keeping the nucleobase concentration low and the mixture at a low reaction temperature increased the yield of 2′-deoxyribonucleoside relative to the amount of added nucleobase, indicating that energy was efficiently generated from glucose via the yeast glycolytic pathway under these reaction conditions. Using a one-pot reaction in which small amounts of adenine, adenosine, and acetone-dried yeast were fed into the reaction, 75 mM of 2′-deoxyinosine, the deaminated product of 2′-deoxyadenosine, was produced from glucose (600 mM), acetaldehyde (250 mM), adenine (70 mM), and adenosine (20 mM) with a high yield relative to the total base moiety input (83%). Moreover, a variety of natural dNSs were further synthesized by introducing a base-exchange reaction into the process.

**Conclusion:**

A critical common issue in energy-requiring bioprocess is fine control of phosphate concentration. We tried to resolve this problem, and provide the convenient recipe for establishment of energy-requiring bioprocesses. It is anticipated that the commercial demand for dNSs, which are primary metabolites that accumulate at very low levels in the metabolic pool, will grow. The development of an efficient production method for these compounds will have a great impact in both fields of applied microbiology and industry and will also serve as a good example of a microbial platform for energy-requiring bioprocesses.

## Background

**Figure 1:**
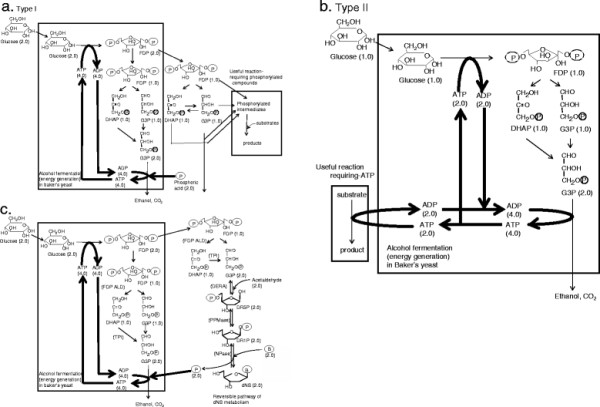
**Concept of energy-requiring bioprocess. a)** Type I; FDP itself or phosphorylated intermediates (eg. G3P, DHAP, etc.) these are generated from glucose serve as substrates for reactions requiring high-energy phosphorylated compounds (e.g., aldol condensation [7,8]). The bold line indicates efficient phosphate take-in process for ATP regeneration. Parentheses represent the number of molecules. **b)** Type II; ATP generated by baker’s yeast served as energy for the reaction requiring ATP. The bold line indicates efficient phosphate take-in process for ATP regeneration. Parentheses represent the number of molecules. **c)** An example of energy-requiring bioprocesses: Microbial production of dNS from glucose, acetaldehyde, and a nucleobase. In this process, FDP generated from glucose by baker’s yeast [4,9] serves as a substrate for fructose 1,6-diphosphate aldolase (FDP ALD) reaction in *E. coli* (DERA-PPMase−co-expressing *E. coli*), and then the generated triose phosphates (DHAP and G3P) are converted to dNS through reactions catalyzed by triose phosphate isomerase (TPI) [10] and the enzymes involved in dNS metabolism (DERA-PPMase-NPase) [9,11,12]. This process is classified into type I in Figure 1a. The bold line indicates efficient phosphate take-in process for ATP regeneration. Parentheses represent the number of molecules.

Enzymatic transformation is an environmentally friendly production process. Industries have attempted to use bioprocesses involving enzymatic transformation to produce chemicals; however, most of the enzymes that have been successfully developed are simple enzymes, such as hydrolases and hydratases. To expand the variations in enzyme-catalyzed processes, a combination of enzyme-catalyzed reactions and their accessory components, such as a cofactor regeneration system, was recently examined. When developing an efficient bioprocess, it is important to consider how to supply the cofactor that accelerates the enzymatic reaction. One good example is enzymatic carbonyl reduction for chiral alcohol synthesis with the cofactor (NAD(P)H) regeneration system [1-3]. We tried to extend this concept to energy-requiring bioprocesses using ATP or phosphorylated high-energy substrates (Figure 1a, b). In this method, two features of baker’s yeast serve as driving force. 1) The excellent ATP regeneration system. 2) Baker’s yeast cell treated by organic solvent (toluene or acetone) temporarily accumulate fructose 1,6-diphosphate (FDP) out of a cell under existence of glucose and phosphate [4-6]. In order to construct a microbial platform for an energy-requiring bioprocess, we established an efficient enzymatic process in which 2′-deoxyribonucleosides (dNSs) are synthesized from glucose, acetaldehyde, and a nucleobase via a C−C coupling reaction requiring a high-energy substrate provided by the glycolytic pathway as an energy-generating system (Figure 1c).

The demand for dNSs is expected to grow as PCR becomes more wide-spread and as the number of new medical technologies using antiviral nucleoside analogs as well as antisense DNA for cancer therapy increase. The examples of dNS derivatives are dNTPs for PCR tool and azidothymidine (AZT) for antiviral drug. However, currently the major sources of 2′-deoxyribonucleoside/2′-deoxyribonucleoside 5-phosphate are hydrolyzed herring and salmon sperm DNA. These sources may not allow us to meet future demands for DNA because of low productivity and production cost limitations. Microbial processes could remove this bottleneck in the dNS supply because cheap and large-scale microbial productions have already been shown to produce many structurally complex compounds on the scale of tons.

Microbial production for industry has already been established with processes for the production of various amino acids, fatty acids and nucleosides such as 5′-inosinic acid [13] and 5′-guanylic acid [14]. In addition, several fermentative production processes for primary metabolites have already been established; however, fermentative production of dNS (or DNA) has not been established yet. Although previous studies have shown that microorganisms can mediate DNA fermentation from sugar [15] and acetic acid [16], the productivity of these processes is impractical. The difficulty of controlling the amount of DNA in cells results in low DNA production with *de novo* synthesis. On the other hand, previous studies reported reductive synthesis from ribonucleotides using purified enzymes [17,18]. However, the substrate, ribonucleoside 5′-triphosphate, used in this process is rather expensive. Based on these considerations, one purpose of this field is to establish an efficient dNS production process with inexpensive materials.

We focused on the enzymatic production of dNS through the reversible dNS degradation pathway catalyzed by deoxyriboaldolase (DERA), phosphopentomutase (PPMase), and nucleoside phosphorylase (NPase) (Figure 1c) [4,9-12]. The process involves an energy-requiring reaction, which is the C−C coupling of the high-energy substrate, D-glyceraldehyde 3-phosphate and acetaldehyde catalyzed by DERA. Our success in enzymatically producing dNSs is the first example in which dNSs were produced from inexpensive materials, such as glucose, acetaldehyde, and a nucleobase.

In this study, we evaluated the one-pot production of dNS using baker’s yeast (energy-generator, phosphorylated-sugar supplier), DERA-PPMase−co-expressing *E. coli*, and commercial purine nucleoside phosphorylase (PNPase). We believe this process is a good example upon which to establish a platform technology for energy-requiring bioprocesses using commercially available baker’s yeast as an energy generator from an inexpensive material, glucose.

## Results

### Characterization and optimization of one-pot dNS production

We characterized and optimized one-pot dNS production using acetone-dried yeast, DEAR-PPMase−co-expressing *E. coli*, and commercial PNPase as catalysts.

#### Construction of DERA-PPMase−co-expressing E. coli, BL21/pACDR-pTS17

As a specific catalyst for this dNS-producing process, we constructed an *E. coli* strain co-expressing DERA-PPMase, *E. coli* BL21/pACDR-pTS17. Protein expression was confirmed by SDS-PAGE analysis of cell-free *E. coli* extracts. Two specific bands with molecular weights of approximately 42,000 and 28,000, which correspond to the size of the T7-tagged *E. coli* PPMase [12] and *K. pneumoniae* B-4-4 DERA [9], were detected. The specific activities of PPMase and DERA were respectively 7.0- and 9.8-fold higher than that of the host strain, *E. coli* BL21.

#### Base specificity

We investigated the nucleobase specificity in the one-pot reaction using adenine, guanine, xanthine, 2,6-diaminopurine, thymine, uracil, and cytosine. These reactions were carried out using 100 mM of each base. The highest dNS production was observed when adenine was used as a substrate. However, the produced dNS was not 2′-deoxyadenosine (dA) but 2′-deoxyinosine (dI, 34.3 mM), which is a deaminated product of dA that is catalyzed by *E. coli* adenosine deaminase*.* Using guanine, 2,6-diaminopurine, the corresponding dNSs were produced with 8.2 mM and 12.3 mM, respectively. Further investigation was carried out with adenine as a base source.

#### Catalyst amounts and stability

The effects of the amount of catalyst (acetone-dried yeast, *E. coli* BL21/pACDR-pTS17, and commercial PNPase) were examined in the one-pot reaction. With acetone-dried yeast cells and wet *E. coli* BL21/pACDR-pTS17 cells, dI production increased with increasing concentrations up to 4% (w/v) and 15% (w/v), respectively, and then decreased when the concentration of cells exceeded those values. In the one-pot reaction system, *E. coli* host cells resulted in greater contamination of unnecessary activities (non-specific phosphatases, nucleosidases, etc.) than baker’s yeast. On TLC analysis of one-pot reaction mixture, 2′-deoxyribose that is de-phosphorylated product of DR5P and dNS by phosphatase and nucleosidase was detected (data not shown). With commercial PNPase, dI production increased as the PNPase concentration increased up to 30 U/ml. A higher PNPase concentration was required to pull the reaction toward dNS synthesis in the one-pot reaction system. A longer incubation resulted in decreased dNS production in this system. Therefore, the feeding effects of the catalysts were examined. When acetone-dried yeast were fed into the reaction at 20 h, the decrease in dNS accumulation was repressed. Thus, baker’s yeast should be fed into the reaction vessel for extended reaction times to maintain sustainable energy generation from glucose. As for DERA-PPMase−co-expressing *E. coli* and commercial PNPase, it was not necessary to feed the catalysts during reactions lasting 30 h.

#### Substrate concentrations

**Figure 2:**
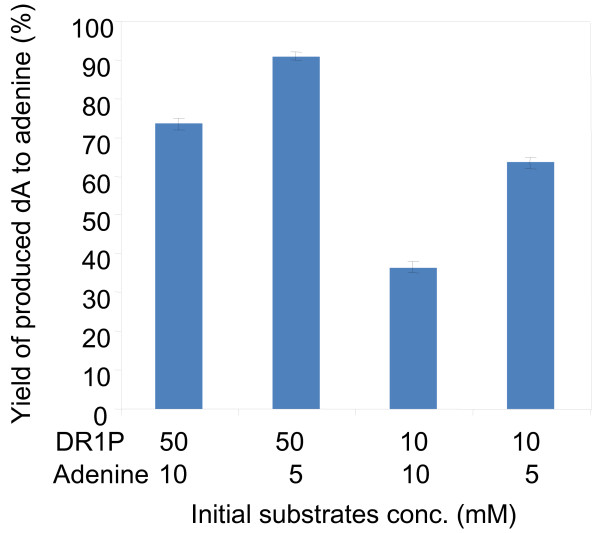
**dA synthesis from DR1P and adenine using commercial PNPase.** These 0.75-ml reaction mixtures contained 50 mM or 10 mM DR1P, 10 mM or 5 mM adenine and 20 mM potassium phosphate buffer (pH 7.0). The reaction was incubated at 20°C for 17 h with shaking (120 rpm). The concentrations of DR1P and adenine in the reaction mixture are indicated in the figure. Three separate experiments were performed. Bars depict the average dA yield relative to added adenine. Error bars depict the standard deviations.

Acetaldehyde: The effects of acetaldehyde concentrations ranging from 0 to 900 mM were examined. dI production increased as the acetaldehyde concentration increased up to 300 mM and then decreased when the acetaldehyde concentrations exceeded 300 mM. In addition, we examined the effects of acetaldehyde feeding. When 50 mM, 150 mM, 300 mM or no acetaldehyde was fed into the reaction at 22 h and 44 h, it was determined that 50 mM was the best acetaldehyde concentration for dNS production. Adenine: The effects of adenine concentrations were also examined. First, we focused on the final step of this system, that is, 2′-deoxyadenosine (dA) synthesis from 2-deoxyribose 1-phosphate (DR1P) and adenine catalyzed by PNPase (Figure 2). The reactions were performed with 10 or 50 mM of commercial DR1P and 5 or 10 mM of adenine using commercial PNPase. dA production proceeded well in the presence of a small amount (5 mM) of adenine and an excess amount (50 mM) of DR1P. This result suggests that the ratio of the adenine concentration to phosphorylated intermediates is an important factor for dNS production. Thus, it might be necessary to keep the adenine concentration much lower than that of DR1P to improve the yield of dNS relative to adenine in the one-pot production system. Then, the effects of the initial adenine concentration were examined in one-pot dNS production from glucose, acetaldehyde, and adenine. The yield of dI to adenine was maintained more than 80% up to 30 mM of adenine but decreased when the initial adenine concentration was higher than 30 mM. Phosphate: dI production increased with increasing initial concentrations of phosphate up to 20 mM, but then decreased at higher concentrations. This tendency was observed in both the early and later stages of the one-pot reaction.

#### Temperature

As for the reaction temperature, we broadly examined the effects of temperatures ranging from 20−50°C using a water bath incubator. The reaction proceeded well at 20°C. Thus, we examined lower temperatures ranging from 5−25°C and found that the reaction proceeded well at 10°C. However, when the reaction was performed in an air incubator at 10°C, the reaction did not proceed at all. This may be due to the fact that the real temperature of the reaction mixture could not be controlled by the air incubator due to the release of fermentative heat by yeast cells. Finally, we decided to perform the reaction at 10°C using a water bath incubator.

### Preparative production of dNS under optimized conditions

**Figure 3:**
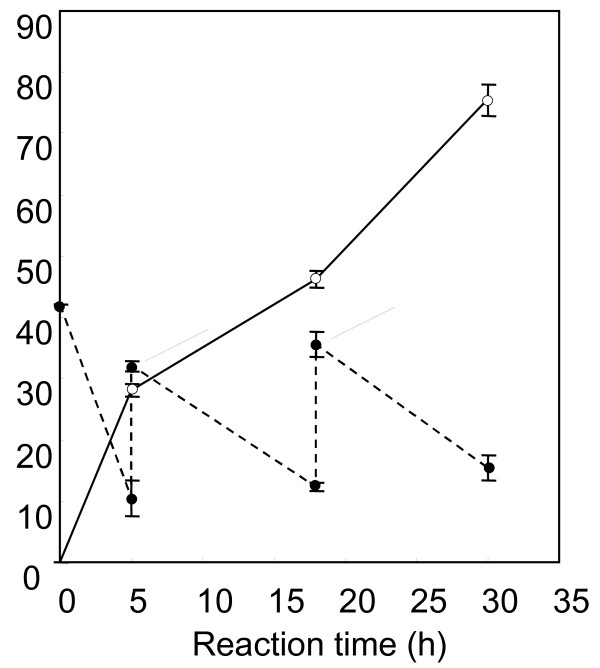
**Time course of dNS production under optimized reaction conditions.** The initial 10.5-ml reaction mixture contained 600 mM glucose, 250 mM acetaldehyde, 30 mM adenine, 25 mM MgSO_4_·7H_2_O, 20 mM potassium phosphate buffer (pH 7.0), 1.0 mM MnCl_2_·4H_2_O, 0.1 mM glucose 1,6-diphosphate, 0.4% (v/v) polyoxyethylenelaurylamine, 1.0% (v/v) xylene, 10 mM adenosine, 4% (w/v) acetone-dried yeast, 15% (w/v) wet *E. coli* BL21/pACDR-pTS17 cells, and 30 U/ml commercial PNPase. The reaction was performed in pH 6.8 (without adjusting), 10°C for 30 h with shaking (120 rpm). During the dNS-forming reaction, 10 N NaOH was used as the alkali solution to adjust the pH to approximately pH 7.5 at 5 h and 18 h. In addition, 0.8% (w/v) acetone-dried yeast, 5 mM adenosine, 25 mM adenine, and 90 mM acetaldehyde were added at 5 h and 18 h. The arrowheads indicate these feeding points. Three separate experiments were performed. Open circles depict the average dI production. Closed circles with the dotted line depict the averages of the sum of the residual nucleosides (adenosine and inosine) and nucleobase (adenine and hypoxanthine). Error bars depict the standard deviations.

Based on the above results, one-pot dNS production was performed with adenine, adenosine, and acetone-dried yeast feeding (Figure 3). 20 mM adenine, 5 mM adenosine, and 0.8% (wt/vol) dried yeast were added to the reaction mixture at 5 h and 18 h. The adenine concentration was maintained at a low level (less than 30 mM) in the reaction vessel at 10°C, which was controlled using a water bath incubator. The reaction pH was adjusted to pH 7.5 at 5 h and 18 h by adding 10 N NaCl because the pH of the reaction mixture tended to decrease. Under the optimized conditions, 75 mM of dI was produced in 30 h. The molar yields of dI to glucose (600 mM), acetaldehyde (430 mM), and base moieties (sum of 70 mM adenine and 20 mM adenosine) were 12%, 17%, and 83%, respectively.

### Diversification of products

We attempted to diversify the products of this multi-component enzyme catalyzed process (Table 1). Because pyrimidine-dNSs and dA are difficult to produce directly in the one-pot reaction system, a base-exchange reaction using enzymatically prepared dI directly without any purification as the substrate was examined using the deoxyribosyltransferase activity of *Lactobacillus helveticus* JCM 1008 [19]. Using washed *L. helveticus* JCM1008 cells, from the dI prepared with the above one-pot reaction (containing 20 mM of dI in the reaction mixture) and a nucleobase (80 mM of adenine, thymine, or cytosine), the corresponding dNSs (dA, dT and dC) were synthesized at 15 mM, 1.5 mM, and 2.1 mM, respectively. The difference in productivities might result from substrates specificity of deoxyribosyltransferase.

**Table 1:** Conversion of enzymatically prepared dl to another dNSs by using deoxyribosyltransferase activity of *L*. *helveticus*

**Substrate base (each 80 mM)**	**Corresponding dNS (mM)**
adenine	14.7(±0.1)
thymine	2.4(±0.1)
cytosine	2.1(±0.1)

These reaction conditions are described in Experimental protocol.

Three separate experiments were carried out. Values are the averages of produced dNS, and values in parenthesis shows standard deviations.

## Discussion

This study established a platform technology for an energy-requiring bioprocess by coupling two types of reactions, an energy-generating reaction and energy-requiring synthesizing reaction. A critical and common issue in energy-requiring bioprocess is tightly controlling the phosphate concentrations [20]. Phosphate consists of energy (ATP, phosphorylated sugars, etc.). Although increased phosphate concentrations are better for energy production, elevated phosphate may negatively affect the progress of the whole process. Here, we tried to resolve this dilemma using an industrially important process, dNS production, as an example of an energy-requiring bioprocess. Glycolysis and the reverse degradation pathway of nucleosides were successfully combined with a step-wise addition of substrates to maintain a suitable concentration of phosphate, and this resulted in efficient energy generation and dNS production.

For dNS production, high concentrations of phosphate were necessary to produce energy. However, these elevated concentrations shifted the reaction equilibrium to dNS degradation and inhibited PPMase [12,21]. Therefore, when investigating energy-requiring bioprocess, it is important to select suitable substrate concentrations and the titer balance of each catalyst in order to increase the taking-in efficiency of phosphate into ATP regeneration and for the efficiency of the entire process.

To prevent PPMase inhibition by phosphorylated compounds [12,21], the initial phosphate concentration was maintained at low levels (20 mM) and a strong taking-in system of phosphate was applied to dNS production by using the energy-generating system of baker’s yeast. When all reactions were performed in the same vessel, in which low concentrations phosphate were efficiently recycled, the dNS production proceeded smoothly. In this system, phosphate generated by PNPase as well as by non-specific phosphatase was recycled effectively even at low concentrations. In addition, by increasing the glucose concentrations, the amount of energy production increased and the equilibrium of whole pathway shifted to dNS synthesis.

Using baker’s yeast as a platform for an energy-requiring bioprocess, it is feasible to practically improve an aldolase-catalyzed process. Aldolases, which catalyze C−C bond formation, have been examined in the synthesis of rare monosaccharides and their derivativies [7,8]. In many useful aldolase reactions, phosphorylated compounds, such as dihydroxyacetone phosphate (DHAP) and D-glyceraldehyde 3-phosphate (G3P), serves as substrates. If energy regeneration by yeast can be introduced into the aldol condensation process as the supplier of energy or high-energy substrates, it can expand the industrial applications. Therefore, this application of microbial glycolysis is a promising strategy to establish a variety of energy-requiring bioprocesses for a sustainable green society.

To date, research on strains with high DNA production and on their culture conditions has been done for the purpose of practical DNA production. Using *Arthrobacter simplex* KY3151 [15] or *P. aeruginasa* KYU-1 [16], 10 mg/L (from glucose) or 7.8 g/L (from acetic acid) of DNA was accumulated in the culture broth, respectively. Based on quantitative comparisons of productivity, our approach exceeds these previous approaches. On the other hand, reductive synthesis of dATP from ATP has also been reported [17,18]. However, this method has the following disadvantages: 1) although ATP can be produced from glucose by fermentative production [22], it will divide ATP production and dATP production into different vessels, 2) purified enzymes are used, and 3) there is a low conversion efficiency for dATP accumulation (40 mM of dATP, 67.8%). Taking these issues into consideration, our process, in which dNS can be directly produced from glucose at high yields relative to base moiety input, is more practical.

One specific problem is the production costs of this process. However, it was important to maintain low adenine concentrations in the reaction mixture to increase the yield of dI to adenine, which is the most expensive material in this process. In addition, maintaining a low reaction temperature, especially when using *E. coli* host cells, could repress unnecessary activities (non-specific phosphatase, nucleosidase, etc.). Controlling all catalyst quantities also seems necessary for efficient progress of the entire energy-requiring bioprocess. As for the stability of catalysts, acetone-dried yeast could not maintain their activity for more than 20 h. This result indicated that some enzymes involved in glycolysis or ATP regeneration in baker’s yeast lost their activity within 20 h. Thus, feeding a small amount of adenine, adenosine, and acetone-dried yeast into the reaction mixture increased dNS production to 75 mM (dI produced, 18.8 g/L), with a high yield (83%) of dI relative to the added base moieties (nucleobase and nucleoside as the energy carrier). These results provide simple and convenient recipe for who want to establish energy-requiring process summarized in Figure 1. It is realizing by input the following materials, acetone-dried yeast, glucose, phosphate, and adenosine with further feeding of acetone-dried yeast and adenosine under phosphate-limited condition at low temperature.

As to the diversity of products, we could produce purine-dNS (dG, dDAP, dI) using the substrate specificity of PNPase in the one-pot reaction. In addition, dA and pyrimidine-dNS (dC, dT) were produced from enzymatically prepared dI using deoxyribosyltransferase activity. Thus, it is possible to produce all natural dNSs with this process. In the future, there will be further demand for dNSs. The nature of DNA, with its repetitive structure and stability, is useful as the raw materials for functional chemicals as well as medicines. Thus, the demand for DNA will expand not only in the fields of biotechnology and medicine but also in chemical material industries. Therefore, it will be necessary to supply efficiently dNS, which are the building block of DNA. We hope our enzymatic process of dNS production with excellently operated energy-requiring process will contribute to these industries by practically and efficiently supplying dNSs.

## Conclusion

As an example of microbial platform for energy-requiring bioprocess, a process for efficient enzymatic synthesis of dNS, in which glycolysis (energy-generation) and reverse degradation pathway of nucleoside (energy-requiring) were successfully combined like an assembly line. A critical common issue in energy-requiring bioprocess is fine control of phosphate concentration. We tried to resolve this problem, and provide the convenient recipe for establishment of energy-requiring bioprocess. If energy regeneration by yeast can be introduced into the aldol condensation process as the supplier of energy or high-energy substrates, it can expand the industrial applications. Therefore, this application of microbial glycolysis is a promising strategy to establish a variety of energy-requiring bioprocesses for a sustainable green society.

In the trend for reproduction and sustainability to be needed, the development of an efficient production method for dNSs, which are primary metabolites that accumulate at very low levels in the metabolic pool but are anticipated to grow in commercial demand, have great impact in both the fields of applied microbiology and industry and will serve as a good example of a microbial platform for energy-requiring processes.

## Methods

### Preparation of acetone-dried yeast

Acetone-dried baker’s yeast cells were prepared according to the method reported by Tochikura et al. [5,6].

### Preparation of DERA- and PPMase−co-expressing *E. coli* transformants

The *Bam*HI-*Eco*RI fragment of pTS8 [9] containing the IPTG-inducible *Tac* promoter and the full-length DERA gene from *K. pneumoniae* B-4-4 [10] was purified by agarose gel electrophoresis. This purified fragment was blunted using a DNA Blunting Kit (TaKaRa Shozo, Kyoto, Japan). The blunted fragment containing the *Tac* promoter and DERA gene was inserted into the *Sma*I-site of pACYC177 carrying a kanamycin resistance gene (NIPPON GENE, Toyama, Japan), yielding the DERA expression plasmid, pACDR. Plasmid pTS17 [12,23], which encodes *E. coli* PPMase, was constructed as previously described. This plasmid contains the ampicillin resistance gene. The DERA expression plasmid, pACDR and PPMase expression plasmid, pTS17 were co-transformed into *E. coli* BL21 cells (Novagen, Darmstadt, Germany). Both ampicillin- and kanamycin-resistant strains were selected, and designated *E. coli* BL21/pACDR-pTS17. *E. coli* transformants were cultivated in Luria−Bertani medium (LB; 1% peptone, 0.5% yeast extract, and 1% NaCl) supplemented with 100 μg/ml ampicillin and 25 μg/ml kanamycin, and 1.0 mM IPTG at 28°C for 24 h. To prepare cell-free *E. coli* extracts, cells were harvested and then disrupted by sonicating in 20 mM Tris/HCl buffer (pH 7.5). After centrifugation (10,000 x *g*, 10 min, at 4°C), the resulting supernatants (approximately 7.5 μg of protein) for each sample were subjected to sodium dodecylsulfate-polyacrylamide gel electrophoresis (SDS-PAGE) as previously described to check the expression of the protein of interest [9].

### DERA activity assay

DERA activity in cell-free extracts was examined based on the DR5P-decomposing activity, which was analyzed by monitoring the reduction of NADH through the coupled reactions of DERA and alcohol dehydrogenase (ADH; Oriental Yeast Co., Ltd., Osaka, Japan), which catalyzes acetaldehyde reduction. A molar extinction coefficient of 6,220 M^−1^ cm^−1^ for NADH was used to calculate the specific activity. The reaction mixture consisted of 200 μl and contained 10 mM Tris/HCl buffer (pH 7.5), 0.5 mM NADH, 37.5 U/ml ADH, 25 mM DR5P, and an aliquot of cell-free *E. coli* extract. The reactions were initiated by adding the enzyme solution and then allowed to proceed at 30°C for 5 min. The reduction of NADH was monitored at a wavelength of 340 nm with a SPECTRA MAX 190 (Molecular Devices, USA). One unit of activity was defined as 1 μ mole of NADH decrease per min per mg protein using cell-free extracts of *E. coli* transformants.

### PPMase activity assay

The PPMase activity in cell-free extracts was examined as dNS production coupling PNPase (Sigma, MO, U.S.A.). The reaction volume was 500 μl and consisted of 50 mM DR5P, 15 mM adenine, 12.5 mM Tris/HCl buffer (pH 7.5), 1.0 mM MnCl_2_·4H_2_O, 30 U/ml commercial PNPase, and an aliquot of cell-free *E. coli* extract. The reactions were initiated by adding the enzyme solution and then allowed to proceed at 30°C for 3.0 h, after which they were stopped by the addition of an equal volume of dimethyl sulfoxide. dNS were quantitated by high performance liquid chromatography (HPLC) at 254 nm [column, Cosmosil 5 C18-AR (4.6 x 150 mm, Nacalai Tesque, Kyoto, Japan); eluent, 100 mM NaClO_4_ containing 0.1% (v/v) H_3_PO_4_; flow rate, 1.0 ml/min; temperature, 40°C]. One unit of activity was defined as 1 μ mole of dNS (dA and dI) synthesized per min per mg protein using cell-free extracts of *E. coli* transformants.

### Preliminary reaction conditions for one-pot dNS production

To evaluate the optimal reaction conditions for dNS production, the reactions were performed as follows. The reaction mixture was 1.5−10.5 ml and included 600 mM glucose, 0−900 mM acetaldehyde, 0−100 mM adenine, 26 mM MgSO_4_·7H_2_O, 0−80 mM potassium phosphate buffer (pH 7.0), 1.0 mM MnCl_2_·4H_2_O, 0.1 mM glucose 1,6-diphosphate, 0.4% (v/v) polyoxyethylenelaurylamine, 1.0% (v/v) xylene, 10 mM adenosine, 2−30% (w/v) acetone-dried yeast, 2.5–22.8% (w/v) wet DERA-PPMase−co-expressing *E. coli* cells, and 5−60 U/ml commercial PNPase. The reactions were carried out at 10−50°C for 2−30 h with shaking (120 rpm). dNS production was monitored by HPLC (described above). Polyoxyethylenelaurylamine and xylene were added to increase the permeability of *E. coli* cells to phosphorylated compounds.

### Enzymatic base-exchange reaction with enzymatically prepared dI as a nucleobase acceptor

In this base-exchange reaction, wet *Lactobacillus helveticus* JCM 1008 cells expressing deoxyribosyltransferase-II [19] were used as the catalyst. *L. helveticus* JCM 1008 cells were cultivated anaerobically in MRS medium at 37°C for 24 h. The cells were harvested by centrifugation (8,000 x *g,* 10 min). The reactions were carried out with 25 mM of enzymatically prepared dI, 80 mM of nucleobase (adenine, thymine, or cytosine), and 10% (w/v) of wet *L. helveticus* JCM1008 cells at 37°C for 12 h with shaking (120 rpm). dNS production was monitored by HPLC (described above).

## Abbreviations

dNS, 2′-deoxyribonucleoside; dA, 2′-deoxyadenosine; dI, 2′-deoxyinosine; dDAP, 2′-deoxy-2,6-diaminopurine nucleoside; dC, 2′-deoxycytidine; dT, Thymidine; Pi, Phosphate; DERA, Deoxyriboaldolase; PPMase, Phosphopentomutase; NPase, Nucleoside phosphorylase; PNPase, Purine-nucleoside phosphorylase; FDP, Fructose 1,6-diphosphate; FDP ALD, Fructose-1,6-diphosphate aldolase; TPI, Triose phosphate isomerase; G3P, D-glyceraldehyde 3-phosphate; DR5P, D-2-deoxyribose 5-phosphate; DR1P, D-2-deoxyribose 1-phosphate; ADH, Alcohol dehydrogenase; MRS, de Man, Rogosa, Sharpe.

## Competing interests

The authors declare no competing financial interests.

## Authors’ contributions

NH conceived, designed and performed most of experiments, analyzed data and wrote this paper. JO and SS conceived and designed on this study. TS and TK conducted the experiments. SM and MS assisted with the experiments and associated analyses. SS supervised the project. NH, MH, JS, and JO wrote the manuscript. All authors read and approved the final manuscript.
